# Correlation of elemental hyperaccumulation among the succulent and non-succulent halophytes of Gujarat, India

**DOI:** 10.1038/s41598-023-42980-8

**Published:** 2023-09-29

**Authors:** Suhas Vyas, Govindasamy Agoramoorthy, Kamlesh Gadhvi, Sandip Gamit, Kiran Dangar

**Affiliations:** 1 Department of Life Sciences, Bhakta Kavi Narsinh Mehta University, Junagadh, Gujarat India; 2 College of Pharmacy and Health Care, Tajen University, Yanpu, Pingtung, Taiwan; 3 NM Sadguru Water and Development Foundation, Dahod, Gujarat India

**Keywords:** Ecophysiology, Tropical ecology, Ecology, Plant sciences

## Abstract

This paper presents new data on the salt tolerance and avoidance mechanisms among various groups of halophytes in India. The halophytic flora in general has positive effect of high saline environments on growth and physiology. The coastal area of the Kachchh district in Gujarat includes about 350 km of shoreline along the Gulf of Kachchh. This study presents data on the elemental accumulation mechanisms in soil and halophytic flora (succulent and non-succulents). The halophytes were divided into two groups namely succulent with thick and fleshy leaves and stems as well as non-succulents with thin leaves and stem. The succulent halophytes included species such as *Salicornia brachiata*, *Suaeda fruticosa* and *Suaeda nudiflora*. The non-succulent halophytes include *Aeluropus lagopoides* and *Urochondra setulosa*. Plant parts namely leaves (Phylloclade for *Salicornia*), stems and roots were analyzed during the monsoon season. The results of soil and plant mineral ion contents differed widely across the intertidal zones in the same habitat. Likewise, the intra species have varied in all nutrient levels and salt concentration. The accumulation of elemental concentration was high during the monsoon season in the succulent *Salicornia brachiata*, especially in leaves that showed Na^+^ reaching high up to 7.6 meq g^−1^, whereas Cl^−^ was noted to be 4.34 meq g^−1^. In the non-succulent halophytes, the accumulation of mineral ion concentration was lower when compared to succulent plants.

## Introduction

Soil salinity continues to threaten the productivity of the land across the world. Scientists have predicted that the salinity will impact over half of the total arable land globally by 2025 leading to a 70% increase in the demand for food recourses^1^. The total land area impacted by salinity has been estimated at about 1125 million hectares^2^. Now in current situations, the coastal agricultural regions face several problems. Globally soil salinization is a scourge for agricultural productivity, crops grown on saline soils suffer on account of high osmotic stress, nutritional disorders and toxicities, poor soil physical conditions and reduced crop productivity^3^. In Bangladesh, some less positively use salt-affected land as shrimp culture-led land use activity, whereas another farmer handles this problem by applying lime, gypsum, etc.^4^. In India, Northern–west regions include, Hariyana, Punjab, Rajasthan (desert regions) and the coastal belt of Gujarat suffering from salinity problems. The coastal belt of Kachchh is primarily composed of sodium (Na^+^) in comparison to calcium and magnesium and the majority of the coastal belt is sodic in nature within 5 km of the shoreline, with the exception of some parts of Mandvi and Mundra taluka^5^. The toxicity of sodium and chloride is predominant in the soil around coastlines^6,7^. Overexploitation and unrestricted deeper drilling, which increase coastal salinity and allow seawater intrusion, are the main causes in the area. The use of this water frequently has a negative impact on human health, agriculture, and soil quality^8^. Some good approaches to utilize this land as growing medicinal plants and algal cultivation that performed and yield well under saline irrigation^9^.

Nevertheless, halophytes are suitable plant candidates for the phytoremediation phenomenon^10^. One of the succulent halophyte *Suaeda fruticosa* has the ability to tolerate lead and zinc metals, in the presence of trace metal elements in its root portion, it has basic characteristics to tolerant high capacity of phytostabilization of trace metal elements in its below-ground structures^11^. Halophytes have adaptations to survive in extreme salinity. Steiner^12^ has classified halophytes under three groups, (1) succulent halophytes with special characteristics to accumulate salt in cell sap and increase succulence, (2) non-succulent halophytes with special salt-secreting gland, and (3) the accumulating halophytes which do not have any kind of mechanisms for salt removal so they accumulate high concentration of salts till death. Some physiologically active molecules play a key role in the saline environment such as proline^13^. In grass *Halopyrum mucronatum* Ca^+2^ is reduced when accumulation of Na^+^ increased^14^. According to Khan^15^, the water and osmotic potential of the halophytic plant *S. fruticosa* becomes more negative with increasing salinity; they also noted that Ca^+2^, Mg^+2^ and K^+^ concentration decreases when salinity increases in the leaf. The presence of Na^+^ and Mg^+2^ content is responsible for alleviating the effects of K^+^. Under the higher concentration of Na^+^, Mg^+2^ and Ca^+2^ reported a decrease in K^+^ content, K^+^ in concentration is high or when the rest of the cation decrease^16^. According to Marschner^17^, K^+^ and Mg^+2^ play parallel key roles for the control of osmoregulation, enzyme activation and cellular pH. Succulent halophytic plants accumulate more salt than that non-succulent plants in their foliar organs. Results also reveal that the non-succulent plant can store a high amount of Ca^2+^, Mg^+2^ and K^+^ in all parts compared to succulent plants. The leaf and stem of the succulent plant have a high amount of Na^+^, S^−2^ and Mg^+2^ when there is a low concentration of Ca^2+^ and K^+^^18^. *Salicornia fruticosa* accumulates a high amount of Na^+^, *Cakile maritima* tissues have the highest values of Ca^+2^, P^−^ and S^−2^. Another finding *C. minimum* showed Na^+^ ion accumulation higher when salinity increases and it directly affects Ca^+2^ and Mg^+2^ low accumulations^19^. The current research focused on salinity variation in the different coastal areas of Kachchh district with salt-accumulating plants, the future scope of the current research is to the reclamation of coastal salt-affected agriculture regions by selected halophytic groups.

## Materials and methods

### Study area

**Figure 1:**
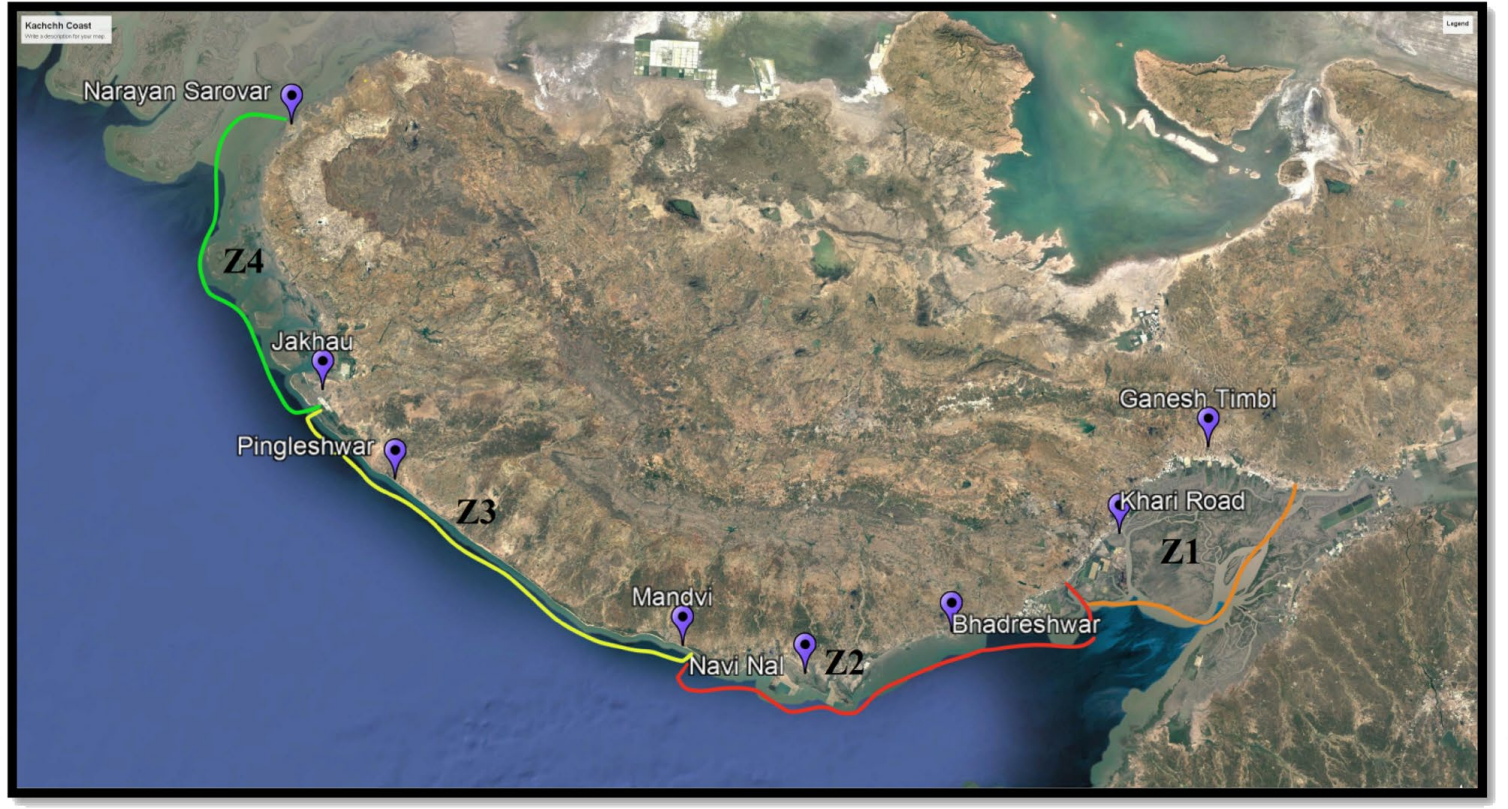
Coastal belt of Kachchh, Gujarat, India. The image was from Google Earth Pro 7.3.3 (https://www.google.com/intl/en_uk/earth/versions/#earth-pro).

Kachchh district consists of approximately 350 km coastal belt out of 1600 km total coastline of Gujarat. Different types of coastal habitats were surveyed and selected for the study. An appropriate number of soil and plant samples were collected from the selected locations during the monsoon season*.* For sample collection coastal area of Kachchh district was divided into four zones, based on the intertidal area viz., zone 1 (20 km), zone 3 (5 km), zone 2 (100 m) and zone 4 (15 km) of the intertidal area^20^ (Fig. 1).

### Permission statement

Selected sites (for the collection of samples from the coastal belt of Kachchh district) are not covered under any protected area or private land. Therefore permission is not required.

### The experimental works

#### Sample collection

Experimental research and field studies on plants including a collection of plant materials according to the Indian Biodiversity Act (2002) and methods used for sample collection as per the American Public Health Association, & American Water Works Association^21^. From the selected locations along the coast of Kachchh district, dominant plant samples were collected from natural habitats in the monsoon during 2019. Soil samples (0–30 cm) were collected from each selected site supporting the coastal vegetation. The samples were collected in triplicate numbers and samples were sun-dried, and passed through a 20-mesh sieve before analysis. The plant samples were thoroughly washed to remove dust, mud, and salts and blotted to dryness. Further, it was dried in the oven at 75–80 °C to a constant weight. The dry material so obtained was finely powdered and preserved for further analysis. The samples were once more dried in an oven, before using for analysis of mineral ions.

#### Plant specimen identification

Plant species were identified by Mr. Kamlesh Gadhvi and Dr. Sandip Gamit with the help of Flora of Gujarat state^22^. The voucher specimens (BKNMU 034, 035, 036, 037 and 038) are deposited at the Department of Life Sciences, Bhakta Kavi Narsinh Mehta University, Junagadh, Gujarat.

#### Sample preparation

##### Plant analysis

1 g plant material was taken into a silica crucible, incinerated and ashed in a muffle furnace at a temperature of 450–480 °C. To achieve complete oxidation of the organic matter, about 0.5–1 ml of concentrated nitric acid was added to the crucibles after cooling them to room temperature. The acid was evaporated in a water bath and the crucibles were again placed in the furnace for complete ashing. After cooling at room temperature, 10 ml of 1:1 hydrochloric acid was added to the crucibles containing the ash and was evaporated in a water bath. This was followed by the addition of 20 ml distilled water and the extract was filtered through Whatman filter paper No. 44 with repeated washing and a final volume of 250 ml was made by adding deionized water. The aqueous extract was further used for the estimation of sodium (Na^+^) and potassium (K^+^) by flame photometry (Systronics-130, India), calcium (Ca^2+^) and magnesium (Mg^2+^) by EDTA titration. For chloride (Cl^−^) 1 g of dry material was boiled in 100 ml of deionized water in a water bath for 30 min. After cooling, the extract was filtered, made up to 100 ml in a volumetric flask and the filtrate was used for the estimation of chloride by Argentometric method^23^.

#### Soil analysis

100 gm dried soil in 200 ml distilled water was taken for mineral analysis. Extract of 1:2 (Soil: water) was made up to 250 ml for analysis of Ca^+2^, Mg^+2^, Na^+^ and mineral ions. The pure extract was used for EC and Cl^−^ analysis^24^.

#### Statistical analysis

Primary data of mineral ions were subjected to statistical tools by using SPSS, ANOVA and Spearman's correlation coefficients were performed for observing the significant variation within the parts of each plant as well as between the groups of plants from different zones.

## Results

### Accumulation of elements in succulent plants

#### *Salicornia brachiata* Miq.

*S. brachiata*, a succulent perennial halophytic plant species showed electrical conductivity of 5.95 (mS/cm) of soil supporting the species. The elemental composition in the soil was ranging between 0.19 and 8.47 (meq g^−100^). The highest accumulation of (6.16 meq g^−1^) was noted for Na^+^ in the *phylloclade* of S. *brachiata*, whereas K^+^ 0.14 meq g^−1^ was observed in the stem and roots of this succulent halophytic species. The concentration of these mineral ions followed a sequence of the accumulation ratios in *phylloclade* is, Na^+^ > Cl^−^ > Mg^+2^ > Ca^+2^ > K^+^, in stem and root it is, Na^+^ > Mg^+2^ > Cl^−^ > Ca^+2^ > K^+^. *S. brachiata* accumulates a high amount of salt in phylloclade compared to the rest of the plants (Table 1).

**Table 1:** Elemental composition in sea green bean, *Salicornia brachiata*.

*S. brachiata*	Phylloclade	Stem	Root	Soil
EC	–	–	–	5.95 ± 1.09
				15.80*
Ca^+2^	0.37 ± 0.06	0.25 ± 0.11	0.23 ± 0.10	0.98 ± 0.70
	53.77**	50.86**	12.77*	3298.2^ns^
Mg^+2^	0.86 ± 0.08	0.53 ± 0.16	0.55 ± 0.23	1.38 ± 0.29
	5.87^ns^	37.60**	121.81***	295.81^ns^
Na^+^	6.16 ± 1.33	1.43 ± 1.67	1.14 ± 1.01	8.28 ± 1.60
	50.26**	216.12^ns^	252.87^ns^	27.55**
K^+^	0.17 ± 0.04	0.14 ± 0.02	0.14 ± 0.03	0.19 ± 0.03
	77.6***	7.31*	11.49*	20.61**
Cl^−^	2.50 ± 1.24	0.41 ± 0.33	0.50 ± 0.39	8.47 ± 1.74
	40.38**	186.09^ns^	190.62^ns^	10.04*

(EC; (mS/cm), Ca^+2^, Mg^+2^, Na^+^, K^+^, Cl^−^; (for plant, meq g^−1^ and soil meq g^−100^) Each value of parameter represents mean value of triplicate samples from four zones, mean ± SD and F value of ANOVA is also indicated, when *P* < 0.05).

#### *Suaeda nudiflora* Moq.

*S. nudiflora* plant grows under less saline soil compared to the other succulent halophytes. The EC value of soil is 1.30 (mS/cm), generally the habitat of this plant has low salt concentration. The element concentration in the soil of this plant is between 0.06 and 2.62 (meq g^−100^). The highest accumulated element was Na^+^ 3.87 (meq g^−1^) in the part leaves, whereas K^+^ was in a low amount (0.15 meq g^−1^) in roots. The storage of elements in leaves is followed by this high to a low value, Na^+^ > Cl^−^ > Mg^+2^ > Ca^+2^ > K^+^, in the stem it is, Na^+^ > Mg^+2^ > Cl^−^ > Ca^+2^ > K^+^, wherein the root, only changing Mg ions, Mg^+2^ > Na^+^ > Ca^+2^ > Cl^−^ > K^+^ (Table 2).

**Table 2:** Elemental composition in *Suaeda nudiflora*.

S.* nudiflora*	Leaves	Stem	Root	Soil
EC	–	–	–	1.30 ± 0.24
				2.26^ns^
Ca^+2^	0.44 ± 0.07	0.28 ± 0.01	0.35 ± 0.04	0.34 ± 0.13
	1^ns^	0.48^ns^	10^ns^	304.2**
Mg^+2^	1.47 ± 0.56	0.61 ± 0.23	0.48 ± 0.15	0.30 ± 0.19
	0.13^ns^	10.98^ns^	52.9*	729**
Na^+^	3.87 ± 0.66	0.91 ± 0.15	0.44 ± 0.19	2.09 ± 0.43
	518.71**	9.38^ns^	4.84^ns^	9.34^ns^
K^+^	0.24 ± 1.12	0.17 ± 0.08	0.15 ± 0.02	0.06 ± 0.00
	57.8*	450**	0.10^ns^	0.29^ns^
Cl^−^	1.65 ± 0.56	0.51 ± 0.28	0.34 ± 0.27	2.62 ± 0.69
	166.99**	43,278.95^ns^	29.35*	63.15*

(EC; (mS/cm), Ca^+2^, Mg^+2^, Na^+^, K^+^, Cl^−^; (for plant, meq g^−1^ and soil meq g^−100^) Each value of parameter represents mean value of triplicate samples from four zones, mean ± SD and F value of ANOVA is also indicated, when *P* < 0.05).

#### *Suaeda fruticosa* Forssk. ex J.F.Gmel

*S. fruticosa* was the second-highest salt accumulator plant in current research. EC value of soil was 4.02 (mS/cm), whereas other elements ranging between low to high concentrations of 0.19 to 4.94 (meq g^−100^). The higher accumulated element was Na^+^ which is 4.37 (meq g^−1^) and the lowest was K^+^ 0.14 (meq g^−1^). The mineral accumulation in leaves high to low amount, Na^+^ > Cl^−^ > Mg^+2^ > Ca^+2^ > K^+^, in stem it was, Na^+^ > Mg^+2^ > Ca^+2^ > Cl^−^ > K^+^, when root has, Mg^+2^ > Na^+^ > Cl^−^ > Ca^+2^ > K^+^ (Table 3).

**Table 3:** Elemental composition in Shrubby Seablite, *Suaeda fruticosa*.

S. *fruticosa*	Leaves	Stem	Root	Soil
EC	–	–	–	4.02 ± 3.81
				28.68*
Ca^+2^	0.34 ± 0.13	0.34 ± 0.11	0.27 ± 0.11	0.70 ± 0.69
	800**	200**	115.6**	2452.3***
Mg^+2^	0.59 ± 0.20	0.57 ± 0.11	0.53 ± 0.009	0.84 ± 0.52
	98.76**	655.60**	10.53*	315.26**
Na^+^	4.37 ± 0.33	0.74 ± 0.12	0.37 ± 0.07	4.94 ± 3.69
	1.54^ns^	92.86*	26.98*	2140.1***
K^+^	0.18 ± 0.001	0.14 ± 0.04	0.15 ± 0.01	0.13 ± 0.10
	1.99^ns^	8.05^ns^	4.53^ns^	24.26**
Cl^−^	1.48 ± 0.90	0.29 ± 0.09	0.28 ± 0.13	5.48 ± 3.35
	375.77**	5^ns^	3.46^ns^	12446^ns^

(EC; (mS/cm), Ca^+2^, Mg^+2^, Na^+^, K^+^, Cl^−^; (for plant, meq g^−1^ and soil meq g^−100^) Each value of parameter represents mean value of triplicate samples from four zones, mean ± SD and F value of ANOVA is also indicated, when *P* < 0.05).

### Accumulation of elements in non-succulent plants

#### *Aeluropus lagopoides* (L.) Thwaites

In the current research non-succulent halophytic grass plant *A. lagopoides* showed high salt tolerance. The EC value of soil was (4.89 mS/cm), with other elements ranging between higher to lower 5.86 to 0.08 (meq g^−100^), respectively. Here also a high element accumulator part was leaves same as succulent halophytic plants. Na^+^ was in a higher value of 1.47 meq g^−1^, while K^+^ in the lowest amount was 0.10 meq g^−1^. Major element composition elements-wise, were, Na^+^  > Cl^−^ > Mg^+2^ > Ca^+2^ > K^+^, these ratios followed by leaves. The amount of elements in stem was, Na^+^ > Mg^+2^ > Ca^+2^ > Cl^−^ > K^+^, and in roots was, Na^+^ > Mg^+2^ > Cl^−^ > Ca^+2^ > K^+^. Most of the elements are in a high amount in leaves but in the stem the Mg^+2^ with a high amount of 0.93 (meq g^−1^) while the root is high with the concentration of Ca^+2^ 0.59 (meq. g^−1^) (Table 4).

**Table 4:** Elemental composition in grass, *Aeluropus lagopoides*.

*A. lagopoides*	Leaves	Stem	Root	Soil
EC	–	–	–	4.89 ± 3.16
				101.14**
Ca^+2^	0.52 ± 0.09	0.50 ± 0.28	0.59 ± 0.30	0.90 ± 0.74
	4.99^ns^	37.63**	6.44*	1514.4^ns^
Mg^+2^	0.77 ± 0.24	0.93 ± 0.36	0.71 ± 0.29	1.04 ± 0.26
	1.68^ns^	21.73***	7.54*	8.64*
Na^+^	1.47 ± 0.35	1.16 ± 0.38	0.97 ± 0.53	5.86 ± 2.50
	27.00**	13.44	172.01***	109.91***
K^+^	0.16 ± 0.02	0.14 ± 0.02	0.10 ± 0.03	0.08 ± 0.04
	222.55^ns^	7.6*	69.46*	16.94*
Cl^−^	0.87 ± 0.28	0.49 ± 0.31	0.63 ± 0.60	5.83 ± 3.71
	56.57***	24.86**	446.18^ns^	835.9^ns^

(EC; (mS/cm), Ca^+2^, Mg^+2^, Na^+^, K^+^, Cl^−^; (for plant, meq g^−1^ and soil meq g^−100^) Each value of parameter represents mean value of triplicate samples from four zones, mean ± SD and F value of ANOVA is also indicated, when *P* < 0.05).

#### *Urochondra setulosa* (Trin.) C.E.Hubb

Comparatively, *U. setulosa* accumulates low elements than *A. lagopoides*, but interestingly it growing under a highly saline environment than *A. lagopoides*, the soil of this plant has a high value of Na^+^, Cl^−^, was 6.32 and 6.77 (meq g^−100^) respectively. EC of soil was 5.88 (mS/cm), when other element concentration wise high to low ranged was, 6.77 to 0.12(meq g^−100^). Elements concentration ranges from high 1.19 (meq g^−1^) to low 0.06 (meq g^−1^). The ratios of accumulated elements in leaves, Na^+^ > Mg^+2^ > Cl^−^ > Ca^+2^ > K^+^, whereas in stems, Mg^+2^ > Na^+^ > Ca^+2^ > Cl^−^ > K^+^, these ratio followed by roots. The major ion stored in the stem and root of *U. setulosa* is Mg^+2^ (Table 5).

**Table 5:** Elemental composition in *Urochondra setulosa*.

*U. setulosa*	Leaves	Stem	Root	Soil
EC	–	–	–	5.88 ± 2.36
				124.51**
Ca^+2^	0.54 ± 0.22	0.41 ± 0.39	0.56 ± 0.24	0.94 ± 0.61
	169.36***	1195.36^ns^	292.15^ns^	1514.4^ns^
Mg^+2^	0.86 ± 0.30	0.63 ± 0.45	0.82 ± 0.16	0.91 ± 0.31
	470.17^ns^	3855.46^ns^	53.43**	8.64*
Na^+^	1.19 ± 0.09	0.59 ± 0.20	0.74 ± 0.30	6.32 ± 1.35
	0.06^ns^	17.80**	174.36***	109.91***
K^+^	0.14 ± 0.02	0.092 ± 0.01	0.069 ± 0.02	0.12 ± 0.09
	26.54**	16.43*	154.08***	16.94*
Cl^−^	0.78 ± 0.28	0.39 ± 0.07	0.33 ± 0.04	6.77 ± 1.81
	403.74^ns^	397.82^ns^	28.26**	835.9^ns^

(EC; (mS/cm), Ca^+2^, Mg^+2^, Na^+^, K^+^, Cl^−^; (for plant, meq g^−1^ and soil meq g^−100^) Each value of parameter represents mean value of triplicate samples from four zones, mean ± SD and F value of ANOVA is also indicated, when *P* < 0.05).

### Comparative accumulation of elements in succulent and non-succulent halophytes

**Figure 2:**
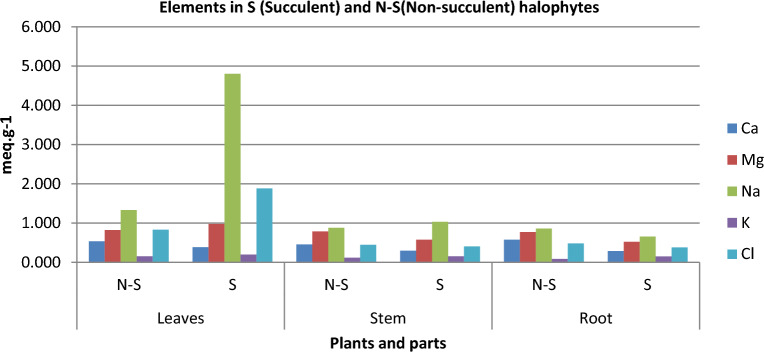
Comparative elements in succulent and non-succulent halophytes among different parts.

Results show among these halophytic groups, elements accumulation in three parts, has been varying at each part. Succulent plants stored the majority of the component in foliar organs when in non-succulent plants is to be in the root. The Mg^+2^ among these both groups of plants, in non-succulent plants accumulation of Mg^+2^ in the stem was high while in succulents plants it was higher in foliar parts (Fig. 2).

### Spearman's correlation coefficient studies

The statistical analysis conducted on the data yielded intriguing findings, unraveling significant interactions among various elements within different parts of succulent and non-succulent halophytic plant species. These discoveries contribute to a deeper scientific understanding of the complex relationships governing these plants' physiological processes. In the succulent plants, an examination of the correlation coefficients revealed a strong positive correlation (0.857**) between K^+^ ions in the leaves and stems. This suggests a tightly coordinated transport mechanism between these plant parts, facilitating the efficient uptake and distribution of potassium ions. Furthermore, a positive correlation was observed between Cl^−^ ions in the leaves and both stem Cl^−^ ions and root Ca^+2^ and Cl^−^. This points to a potential interplay between chloride and calcium ions in maintaining ion homeostasis and osmotic regulation within succulent species.

Interestingly, a positive relation was identified between stem Ca^+2^ and both stem Cl^−^ ions and root Ca^+2^. This implies a potential regulatory mechanism wherein calcium ions may modulate chloride ion transport or compartmentalization. In addition, a positive correlation was detected between stem and root Mg^+2^, indicating a concerted involvement of magnesium ions in essential physiological processes across these plant parts. A particularly noteworthy discovery emerged from the highly significant positive correlation (0.970**) observed between stem Cl^−^ and root Ca^+2^. This association suggests a possible role for chloride and calcium ions in mediating ion transport and cellular signaling pathways crucial for plant growth and adaptation to saline environments. Intriguingly, negative correlations were identified, such as root K^+^ exhibiting an inverse relationship with leaves Cl^−^ (− 0.810*) and both stem (− 0.762*) Mg^+2^ and Cl^−^. This highlights potential antagonistic interactions or regulatory mechanisms between potassium and chloride ions within succulent species (Table 6).

**Table 6:** Spearman's correlation of elements in different parts of succulent halophytes.

Parts	L					S					R					
	Ca^+2^	Mg^+2^	Na^+^	K^+^	Cl^−^	Ca^+2^	Mg^+2^	Na^+^	K^+^	Cl^−^	Ca^+2^	Mg^+2^	Na^+^	K^+^	Cl^−^	
L	Ca^+2^	1.00														
	Mg^+2^	0.45	1.00													
	Na^+^	− 0.14	− 0.12	1.00												
	K^+^	0.36	0.24	0.05	1.00											
	Cl^−^	− 0.21	− 0.19	0.41	− 0.36	1.00										
S	Ca^+2^	0.12	− 0.24	− 0.17	0.00	0.67	1.00									
	Mg^+2^	0.05	0.05	− 0.17	− 0.57	0.55	0.38	1.00								
	Na^+^	− 0.10	0.31	− 0.10	0.43	0.26	0.21	0.07	1.00							
	K^+^	0.43	0.29	0.14	.857**	− 0.67	− 0.43	− .714*	0.05	1.00						
	Cl^−^	0.29	0.06	0.07	− 0.23	.826*	.826*	0.67	0.18	− 0.54	1.00					
R	Ca^+2^	0.29	0.17	− 0.14	− 0.24	.738*	.833*	0.69	0.24	− 0.57	.970**	1.00				
	Mg^+2^	0.31	− 0.19	0.24	− 0.52	0.60	0.33	.714*	− 0.19	− 0.52	0.68	0.57	1.00			
	Na^+^	− 0.38	0.05	0.41	0.00	0.50	0.00	− 0.05	0.69	− 0.19	0.12	0.07	0.05	1.00		
	K^+^	0.00	0.02	− 0.21	0.21	− .810*	− 0.50	− .762*	− 0.50	0.52	− .755*	− 0.69	− 0.69	− 0.48	1.00	
	Cl^−^	− 0.14	− 0.19	0.29	− 0.45	.857**	0.52	0.36	0.24	− 0.67	0.68	0.62	0.60	0.64	− 0.64	1.00

(L; Leaves, S; Stem and R; Root) **Correlation is significant at the 0.01 level, *Correlation is significant at the 0.05 level.

Turning to the non-succulent halophytic group, compelling correlations were uncovered. Leaves Ca^+2^ exhibited strong positive correlations with stem Ca^+2^ (0.929**), Mg^+2^ (0.994**), and root Ca^+2^ (0.810*), suggesting a coordinated regulation of calcium and magnesium ions across various plant parts. This likely reflects the importance of these divalent cations in maintaining cellular integrity, enzymatic activity, and signaling processes in non-succulent halophytes. Remarkably, stem Cl^−^ demonstrated a significant positive correlation (0.786*) with leaves Mg^+2^, while exhibiting a significant negative correlation (− 0.762*) with leaves K^+^. This intricate interplay suggests the existence of complex ion transport systems and compartmentalization mechanisms involving chloride, magnesium, and potassium ions within non-succulent halophytes.

Further analyses unveiled a noteworthy negative correlation trend (− 0.714*) between leaves K^+^ and root Na^+^, indicating potential ion antagonism or cellular regulation between these two elements. Notably, stem Mg^+2^ and Ca^+2^ displayed a highly significant positive correlation (0.922**), implying their co-regulation and shared involvement in diverse metabolic and physiological processes within non-succulent halophytes. Additional investigations shed light on root Na^+^, which exhibited a significant positive correlation with both stem Na^+^ (0.714*) and root Cl^−^ (0.857**). Concurrently, root Cl^−^ displayed positive correlations with stem Na^+^ (0.833*) and root Na^+^ (0.810*). These associations suggest the potential involvement of sodium and chloride ions in osmotic regulation, ion transport, and ionic balance maintenance in the roots of non-succulent halophytes (Table 7).

**Table 7:** Spearman's correlation of elements in different parts of non-succulent halophytes.

Parts	L					S					R					
	Ca^+2^	Mg^+2^	Na^+^	K^+^	Cl^−^	Ca^+2^	Mg^+2^	Na^+^	K^+^	Cl^−^	Ca^+2^	Mg^+2^	Na^+^	K^+^	Cl^−^	
L	Ca^+2^	1.00														
	Mg^+2^	0.48	1.00													
	Na^+^	0.24	0.29	1.00												
	K^+^	− 0.19	− 0.57	0.05	1.00											
	Cl^−^	0.50	0.52	0.48	− 0.64	1.00										
S	Ca^+2^	.929**	0.29	0.33	− 0.12	0.62	1.00									
	Mg^+2^	.994**	0.46	0.29	− 0.20	0.52	.922**	1.00								
	Na^+^	0.19	− 0.07	0.45	− 0.12	0.05	0.12	0.28	1.00							
	K^+^	− 0.05	− 0.52	0.43	0.62	− 0.10	0.10	0.00	0.41	1.00						
	Cl^−^	0.48	.786*	0.48	− .762*	0.67	0.36	0.50	0.45	− 0.33	1.00					
R	Ca^+2^	.810*	0.41	− 0.07	− 0.55	0.48	0.69	.826*	0.24	− 0.36	0.52	1.00				
	Mg^+2^	0.32	0.49	− 0.29	0.06	− 0.04	0.17	0.24	− 0.48	− 0.22	0.02	0.04	1.00			
	Na^+^	0.12	0.43	0.41	− .714*	0.45	0.02	0.18	.714*	− 0.12	.857**	0.31	− 0.30	1.00		
	K^+^	0.60	0.17	0.07	− 0.24	0.57	0.60	0.62	0.14	0.31	0.21	0.57	0.18	0.12	1.00	
	Cl^−^	0.00	0.17	0.57	− 0.29	0.10	− 0.07	0.07	.833*	0.05	0.62	0.10	− 0.55	.810*	− 0.26	1.00

(L; Leaves, S; Stem and R; Root) **Correlation is significant at the 0.01 level, *Correlation is significant at the 0.05 level.

## Discussion

Halophytes are a wonderful group of plants having unique physiological adaptations against extreme saline environments. Physiological adaptation to minimize salt concentrations of the cells or physiological exclusion by root membranes. In principle, salt tolerance is achieved by only salt exclusion or salt inclusion. Marschner^25^, Koyro^26^, gave possible mechanisms of vascular halophytes to adjust to high external NaCl salinity. The ionic compositions of soils revealed that Na^+^ and Cl^−^ were major constituents, with concentrations ranging from 2.09 to 8.47 meq g^−100^ for succulent plant habitat and 5.83 to 6.77 meq g^−100^ for non-succulent plant habitat. It was also observed that Cl^−^ concentrations were higher than Na^+^ concentrations at all habitats. Rogel et al.^27^ found a similar trend for Na^+^ and Cl^−^ in environments, supporting *Arthrocnemum macrostachyum* and *Sarcocornia fruticosa*.

Mineral ion assessment in *Aeluropus lagopoides*, etc., leaves and soil sediment of *A. lagopoides* shows efficient K^+^ uptake and shows maximum Fe^+2^, Mn^+2^, P^−^ and N content than *C. tagal*. Leaves of *A. lagopoides* show high Na^+^ 2.75 and Cl^−^ about 3.64 g/100 g dry weight, it has significant compared to *C. tagal* and *L. racemosa*. The leaves of the majority of halophyte plants accumulate of salt ions and other mineral ions, it is especially stored in foliar organs^28,29^. According to Milić, D^30^, *S. europaea*, *S.maritima* and *S. soda*, above-ground organs of these halophytes accumulate more Na^+^ than Mg^+2^, Ca^+2^ and K^+^, they also revealed that in different habitats these halophytes having more cations in maritime saline areas than inland saline areas. One of the interesting studies revealed by^19^ in the Eu-halophytes succulent and salt-recreating plants shows a high concentration of Na^+^, S^−2^, and Mg^+2^ and a low concentration of Ca^+2^ and K^+^ ions Whereas in pseudo-halophytes, facultative halophytes and eury-hydro-halophytic species, which lack succulent shoots, resulted in low concentration of Na^+^, S^−2^ and Mg^+2^ and high in Ca^+2^ and K^+^ in their leaves, although the study also targets some taxonomic identical families for this kind of elemental variation, in the family, Chenopodiaceae and Plumbaginaceae accumulate high Na^+^ and Mg^+2^, and low Ca^+2^ and K^+^, other families Caryophyllaceae having high K^+^, whereas Poaceae with low Na^+2^. The amount of foliar Ca^+2^ is high in Asteraceae, Boraginaceae and Brassicaceae families.

If the level of salinity increases, it has been observed by Khan^31^, the leaves of perennial halophytes, *S. fruticosa* Ca^+2^, Mg^+2^ and K^+^ concentration decrease. In another stem succulent plant, *H. recurvum,* the stage of succulence increases with low salinity and decreases with high salinity, here changes in different parts of plant-like, in shoot and root values of Ca^+2^, Mg^+2^ and K^+^ content reduce at high salinity. Gulzar^32^, some grass species like *A. lagopoides* and *U. setlosa*, high salt-tolerant non-succulent plants, concluded that it can be cultivated using saline water, and *S. ioclados* is comparatively less salt-tolerant, these plant species growing under its suitable habitat, where brackish water is accessible when water potential and osmotic potential is negative at that time in aerial parts of *A. lagopoides* salinity increases^33^. Joshi, A. J., & Iyengar, E. R. R.^34^ observed accumulation of Cl^−^ in *S. nudiflora* in different seasons viz., monsoon 4.98 meq g^−1^, winter 5.88 meq g^−1^ and summer 5.71 meq g^−1^ and for *S. brachiata,* monsoon 9.83 meq g^−1^, winter 6.85 meq g^−1^ and summer 8.26 meq g^−1^ these results were higher compare to our study. Vyas^35^ noted Cl^−^ in *S. nudiflora* about 8.15 to 12.52 meq g^−1^ these concentrations are also higher than the comparatively present study. Month-wise accumulation of Na^+^ and other ions in *S. brachiata* phylloclade, a high concentration noted in the month of September at about 7.65 meq g^−1^, while the lowest was noted during December at about 6.9 meq g^−1^^36^. Studies on the accumulation of inorganic ions showed that Na^+^ ranged between 5.52 and 8.37 meq g^−1^ in two succulent halophytes *S. brachiata* and *S. fruticosa*^37^, which showed similar kind of observation.

## Conclusion

The present investigation elucidated the mineral ion accumulation profiles of succulent and non-succulent halophytic plant species, showcasing their profound associations with diverse ions. *Salicornia brachiata*, a representative succulent species, exhibited a robust growth in saline, muddy areas, whereas *Sueada nudiflora*, another succulent plant, thrived predominantly in wasteland regions along the Kachchh coast. The salinity levels in all five species primarily stemmed from the contributions of Na^+^, Cl^−^, Mg^+2^, Ca^+2^ and K^+^. Remarkably, our dataset unveiled elevated concentrations of Na^+^ and Cl^−^, whereas K^+^ accumulation remained relatively low within these species. Notably, the surrounding soils of the supporting plants also displayed commensurate high concentrations of these ions.

Of particular interest was the observation of salt avoidance mechanisms in succulent species, in stark contrast to the salt tolerance mechanisms exhibited by non-succulent halophytes. The non-succulent plants effectively excreted salts from their leaves, resulting in the accumulation of ions at low concentrations. Intriguingly, K^+^ content was found to be minimal both in the soil and plant parts, yet it displayed a pronounced affinity for salinity within succulent plants. Furthermore, as the NaCl concentration increased, there was a concomitant decrease in the levels of Ca^+2^, Mg^+2^, and K^+^, as deduced from the outcomes derived from soil samples supporting both succulent and non-succulent halophytic species.

The adaptive propensities of succulent plants manifest as heightened elemental concentrations in their foliar structures. Specifically, our investigations revealed the pronounced accumulation of ions, particularly Na^+^ and Cl^−^, within the Phylloclade of *S. brachiata*. The comprehensive amalgamation of findings from three distinct succulent plant species demonstrated that leaves exhibited significantly greater accumulation of Na^+^, Cl^−^, and Mg^+2^ compared to stems and roots. The total salt content in all five species was predominantly contributed by Na^+^, Cl^−^, Mg^+2^, Ca^+2^ and K^+^, and corresponding high concentrations of these ions were also discernible in the associated soils.

The surplus mineral ion content was selectively allocated to the phylloclade and leaves, primarily due to the presence of adaptive mechanisms that facilitate stress management and facilitate the transformation of excess mineral ions into secondary metabolites, ultimately engendering succulence in these particular organs. The comprehensive correlation study ascertained a statistically significant positive correlation between Ca^+2^ and Mg^+2^ within both groups of plants. Moreover, a positive relationship was discerned between stem and root ions Na^+^ and Cl^−^, while a limited number of negative correlations, particularly involving K^+^, were also identified.

The comprehensive data pertaining to the elemental accumulation patterns across distinct plant parts of succulent and non-succulent halophytic species, as well as within their associated habitats, were furnished for the first time in this study. This pioneering work offers a promising avenue for harnessing the hyperaccumulation capacities inherent to succulent and non-succulent halophytes for phytoremediation endeavors, underscoring their potential utilization in addressing salinity-related challenges afflicting affected ecosystems.

## Data Availability

The datasets used and/or analysed during the current study available from the corresponding author on reasonable request.
